# Respiratory Frequency during Exercise: The Neglected Physiological Measure

**DOI:** 10.3389/fphys.2017.00922

**Published:** 2017-12-11

**Authors:** Andrea Nicolò, Carlo Massaroni, Louis Passfield

**Affiliations:** ^1^Department of Movement, Human and Health Sciences, University of Rome “Foro Italico”, Rome, Italy; ^2^Unit of Measurements and Biomedical Instrumentation, Departmental Faculty of Engineering, Università Campus Bio-Medico di Roma, Rome, Italy; ^3^Endurance Research Group, School of Sport and Exercise Sciences, University of Kent, Kent, United Kingdom; ^4^Faculty of Kinesiology, University of Calgary, Calgary, Canada

**Keywords:** breathing, effort, wearable sensors, training monitoring, athletes

## Abstract

The use of wearable sensor technology for athlete training monitoring is growing exponentially, but some important measures and related wearable devices have received little attention so far. Respiratory frequency (*f*_R_), for example, is emerging as a valuable measurement for training monitoring. Despite the availability of unobtrusive wearable devices measuring *f*_R_ with relatively good accuracy, *f*_R_ is not commonly monitored during training. Yet *f*_R_ is currently measured as a vital sign by multiparameter wearable devices in the military field, clinical settings, and occupational activities. When these devices have been used during exercise, *f*_R_ was used for limited applications like the estimation of the ventilatory threshold. However, more information can be gained from *f*_R_. Unlike heart rate, $\dot{\text{V}}$O_2_, and blood lactate, *f*_R_ is strongly associated with perceived exertion during a variety of exercise paradigms, and under several experimental interventions affecting performance like muscle fatigue, glycogen depletion, heat exposure and hypoxia. This suggests that *f*_R_ is a strong marker of physical effort. Furthermore, unlike other physiological variables, *f*_R_ responds rapidly to variations in workload during high-intensity interval training (HIIT), with potential important implications for many sporting activities. This Perspective article aims to (i) present scientific evidence supporting the relevance of *f*_R_ for training monitoring; (ii) critically revise possible methodologies to measure *f*_R_ and the accuracy of currently available respiratory wearables; (iii) provide preliminary indication on how to analyze *f*_R_ data. This viewpoint is expected to advance the field of training monitoring and stimulate directions for future development of sports wearables.

## Introduction

The large diffusion of wearable devices has stimulated interest in athlete training monitoring, with the aim of maximizing performance, and minimizing the risk of injury and illness (Düking et al., 2016). The development of sport-related technologies is occurring rapidly and is often guided by market forces rather than athlete or scientific needs. In this process, it is not uncommon that technological solutions and measures are available before the sport scientist or practitioner can appreciate their importance, and this can reduce the use of new technologies. Emblematic here, is the example of respiratory frequency (*f*_R_), which may provide a better marker of physical effort compared to traditionally monitored physiological variables. However, despite the availability of unobtrusive wearable devices measuring *f*_R_ with relatively good accuracy, the practice of measuring *f*_R_ during training is not common yet.

## Current applications of respiratory wearables

For a long time, *f*_R_ has received little consideration also in the clinical field, despite being recognized as a vital sign capable of predicting serious adverse events. A series of papers entitled “Respiratory rate: the neglected vital sign” (Cheng et al., 2008; Cretikos et al., 2008; Gandevia and McKenzie, 2008; Steichen et al., 2008) and “Rate of respiration: the forgotten vital sign” (Parkes, 2011) contributed to redirect attention to *f*_R_ in the clinical field. These contributions also inspired the present manuscript, which aims to draw attention to the potential of *f*_R_ for monitoring training in sport. Due to its importance as a vital sign, *f*_R_ is currently measured by unobtrusive multi-parameter wearable devices mainly in the military field, clinical setting, and during occupational activities. When these devices have been used during exercise, *f*_R_ is typically used for limited applications such as the estimation of the ventilatory threshold during incremental exercise (Hailstone and Kilding, 2011). Whilst, the disproportionate and progressive increase in *f*_R_, which begins with attainment of the first ventilatory threshold, may be used as a practical non-invasive method for estimating the ventilatory thresholds (Cross et al., 2012), there are other important reasons why athletes should consider monitoring *f*_R_ during training.

## Respiratory frequency as a marker of physical effort

*f*_R_ is often measured in exercise physiology as one of the two components (together with tidal volume) of minute ventilation. However, minute ventilation has typically received much more attention than its components, being the best single indicator of the ventilatory output. Nevertheless, recent evidence suggests that *f*_R_ and tidal volume are regulated by different inputs during exercise, and that their differential responses contain valuable information (Nicolò et al., 2017a,b). *f*_R_ plays an important role during exercise as a strong marker of physical effort, more so than other traditionally monitored physiological variables. The non-linear increase of *f*_R_ during incremental exercise parallels the well-known time course of blood lactate (La^−^), resembling the change in physical effort and task difficulty experienced at exercise intensities above the first ventilatory threshold. In fact, *f*_R_ better reflects physical effort than La^−^ when an incremental test is performed after exercise-induced muscle damage (Davies et al., 2011) or glycogen depletion (Busse et al., 1991), and in patients with McArdle's disease (Voduc et al., 2004). This suggests that physical effort is more causally linked with *f*_R_ than La^−^.

Unlike $\dot{\text{V}}$O_2_, heart rate (HR) and La^−^, *f*_R_ shows an effort-like response during a variety of exercise paradigms. During both time-to-exhaustion and self-paced time trial protocols, *f*_R_ increases approximately linearly over time and approaches maximal values at the end of exercise. This response is observed during both continuous (Nicolò et al., 2016a) and intermittent (Nicolò et al., 2014a,b, 2017b) exercise of different duration, and with a variety of experimental interventions that affect performance. Moreover, unlike other physiological variables, the time course of *f*_R_ is closely associated with that of Rating of Perceived Exertion (RPE) (Nicolò et al., 2014a, 2016a, 2017b). This association is found even after locomotor muscle fatigue (Marcora et al., 2008) and damage (Davies et al., 2009), inspiratory (Mador and Acevedo, 1991) and expiratory (Taylor and Romer, 2008) muscle fatigue, muscle glycogen depletion (Busse et al., 1991), increases in body temperature (Hayashi et al., 2006), hypoxia (Koglin and Kayser, 2013), ingestion of sodium bicarbonate (Robertson et al., 1986), prior endurance exercise (Spengler et al., 2000), and after expiratory muscle training (Suzuki et al., 1995). Conversely, HR, $\dot{\text{V}}$O_2_, and La^−^ are partially dissociated from RPE under some of these experimental interventions. Therefore, *f*_R_ appears to be sensitive to different fatigue states, and thus may present potentially important implications for training and recovery monitoring. Furthermore, *f*_R_ may be a good predictor of time to exhaustion during constant-workload trials (Pires et al., 2011a,b) and can help understand how effort is distributed during self-paced time trials (Nicolò et al., 2014a, 2016a). The observation that *f*_R_ is a stronger correlate of RPE than other physiological variables is not novel (Noble et al., 1973; Robertson et al., 1986), and it has previously been proposed as a variable to monitor during training (James et al., 1989; Neary et al., 1995). However, the importance of *f*_R_ as a marker of physical effort has emerged from recent investigations (Nicolò et al., 2014a, 2016a, 2017b).

**Figure 1:**
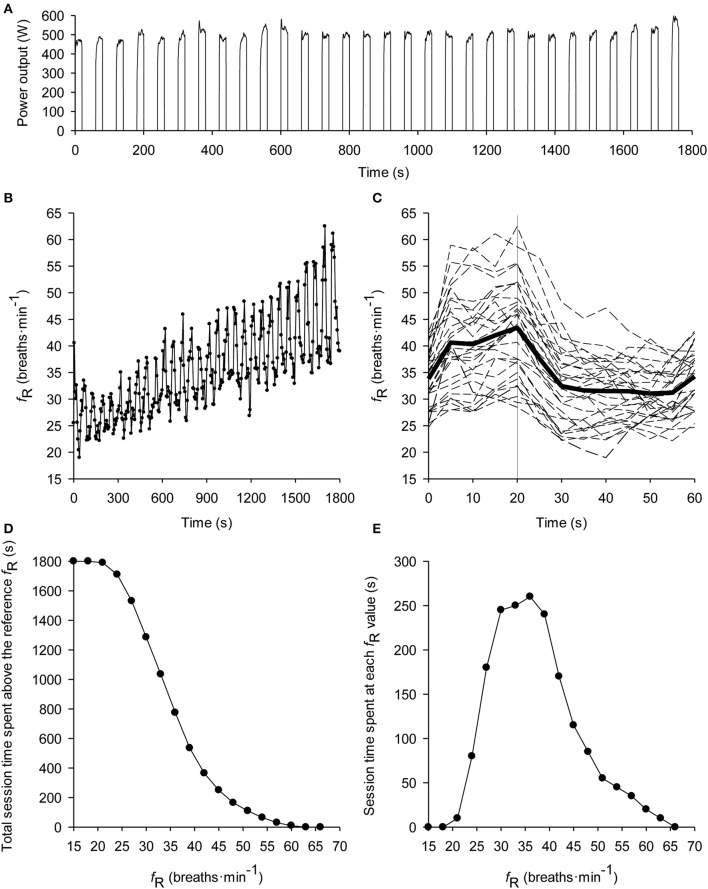
Typical subject performing a 20-s work 40-s rest self-paced intermittent cycling time trial lasting 30 min (i.e., 30 repetitions). Data are from Nicolò et al. (2014a). The time course of power output is depicted in **(A)**. Of note, *f*_R_ responds very fast to the alternation of the work and recovery phases, and increases progressively over time **(B)**. The rapid change in *f*_R_ according to variations in workload can be better observed by showing the time course of *f*_R_ within the 60-s work-recovery cycle **(C)**. The solid thick line represents the average of the entire trial, the dashed lines represent each repetition and the solid vertical line separates the 20-s work from the 40-s recovery. For details on this analysis see Nicolò et al. (2014b). This is also a convenient representation to show *f*_R_ data real time during HIIT. In order to synthesize the effort of the training session, the *f*_R_ distribution **(D)** and concentration **(E)** profiles have also been constructed. The distribution profile describes the time spent above each *f*_R_-value, while the concentration profile describes the time spent at each *f*_R_-value. Both analyses can also be used to describe several training sessions. See Kosmidis and Passfield (2015) for more details on the two analyses.

An important feature differentiating *f*_R_ from other physiological variables is the very fast response at exercise onset and offset. During sustained all-out exercise, *f*_R_ increases rapidly at the beginning of exercise and quickly reaches maximal values that are maintained throughout the trial, even where an exponential decrease in power-output occurs (Nicolò et al., 2015). A rapid response of *f*_R_ is also observed during the alternation of work and recovery phases characterizing high-intensity interval training (HIIT) (Nicolò et al., 2014b, 2017b). Furthermore, *f*_R_ changes in proportion to workload variations in work and recovery across different HIIT sessions (Nicolò et al., 2017b). This makes *f*_R_ a useful variable to describe the fast changes in effort that characterize HIIT (Figures 1A–C). In contrast, $\dot{\text{V}}$O_2_ and HR do not respond abruptly to such changes in workload (Nicolò et al., 2014b, 2017b).

The experimental evidence for *f*_R_ as a marker of effort is substantiated by our understanding of the mechanisms underlying its regulation. One of the major regulators of ventilation during exercise is central command (Forster et al., 2012), i.e., the central neural drive associated with voluntary motor effort. Moreover, it has been suggested that central command regulates preferentially *f*_R_ rather than tidal volume (Nicolò et al., 2017b). Central command is also the sensory signal for perceived exertion (Marcora, 2009), and this provides a neurophysiological explanation for the association observed between perceived exertion and *f*_R_. This is why in the present manuscript we refer to “physical effort” as a theoretical construct which is distinct from, but linked to, perceived effort. Physical effort can be defined as the degree of motor effort, (i.e., the magnitude of central command) (Nicolò et al., 2016b). For the applied sport scientists and practitioners, physical effort (and thereby *f*_R_) reflects how hard, heavy and strenuous a physical task is, whilst perception of effort is the conscious sensation of this physical task (Marcora, 2010).

Sports scientists and practitioners are therefore encouraged to consider *f*_R_ among the variables to monitor in training. Note, most of the evidence suggesting *f*_R_ to be a valid marker of effort comes from studies that used cycling as exercise modality, while less data are available on other exercise modalities. A similar *f*_R_ response was observed during incremental exercise performed either with legs or arms separately as well as with legs and arms combined, despite considerable differences in absolute $\dot{\text{V}}$O_2_, workload and HR (Robertson et al., 1986). This suggests that *f*_R_ reflects the effort exerted during exercise irrespective of absolute workload, metabolic demand, and muscle masses involved. On the other hand, different ventilatory responses have been found when comparing running with cycling (Elliott and Grace, 2010). A different degree of entrainment (coupling between locomotion and breathing rhythms) between cycling and running is often proposed as an explanation for between-modality differences in *f*_R_, but experimental evidence is conflicting. The entrainment phenomenon is well-documented in some sports like rowing, where high inter-individual variability in entrainment pattern is observed (Siegmund et al., 1999). Thus, for rowing a degree of caution is suggested in the interpretation of *f*_R_ until more research is conducted.

## How to measure respiratory frequency in the field

The limited consideration given to *f*_R_ in sport should not be ascribed to technical limitations. It is the easiest ventilatory variable to measure during exercise and several respiratory wearables have been developed. Directly, *f*_R_ can be measured with portable devices registering flow-rate at the mouth (e.g., flow sensors), but require the use of a facemask. These devices (e.g., K5, Cosmed, Rome, Italy) are accurate but relatively obtrusive and not well-suited to training monitoring. However, they are widely used as criterion devices for validating less obtrusive respiratory wearables. Indirectly, *f*_R_ can be measured using the strain and movements of the chest and abdomen induced by ventilation, the sound of breathing, or the effect that ventilation has on biosignals such as electrocardiogram (ECG) and photoplethysmogram (PPG). *f*_R_ can also be measured with sensors monitoring exhaled carbon dioxide, air temperature or humidity, but these sensors are not commonly considered for wearable solutions used in sport.

The majority of commercially-available respiratory wearables register ventilation-induced thoracic and/or abdominal strain through sensors embedded into straps or clothes. Commonly used sensors are inductive (Hexoskin®, Carré Technologies Inc., Montreal, Que., Canada; LifeShirt®, Vivometrics, Inc., Ventura, CA, U.S.A.; Equivital™ EQ02 LifeMonitor™, Hidalgo Cambridge, U.K.), piezo-electric (Pneumotrace II™, UFI, Morro Bay, CA, USA), capacitive (Zephyr™ BioHarness™, Zephyr Technology, Auckland, New Zealand), and piezo-resistive (Wearable Wellness System™, Smartex S.r.l., Italy). The accuracy of most of these respiratory wearables is good as assessed by comparison with a flow sensor criterion device. For instance, a mean average difference (bias) ± limits of agreement (LoA) of ~0.3 ± 2 and 0.2 ± 2.4 breaths·min^−1^ was found for Hexoskin® during submaximal incremental walking (Villar et al., 2015) and for Equivital™ EQ02 LifeMonitor during moderate-intensity walking and running (Liu et al., 2013), respectively. A bias ± LoA of −0.1 ± 5.7 breaths·min^−1^ was found for LifeShirt® during a maximal incremental running test (Witt et al., 2006). A bias ± LoA of −0.6 ± 5 and 0.2 ± 8.3 breaths·min^−1^ was found for Zephyr™ BioHarness™ during a maximal incremental running test and a prolonged moderate-intensity running trial in the heat, respectively (Kim et al., 2013). However, direct comparison of the accuracy of different strain sensors in estimating *f*_R_ during exercise is lacking, and requires further investigation.

Respiratory wearables positioned on the torso can be affected by non-respiratory chest and abdomen movements during locomotion. This problem is commonly addressed when respiratory wearables based on movement sensors are used like accelerometer-based devices registering chest and/or abdomen movements (i.e., inclination changes), and algorithms resilient to motion artifacts have been developed (Liu et al., 2011). Compared to the use of a single accelerometer, the estimation of *f*_R_ improved with a sensor fusion method combining accelerometer and gyro-sensor outputs (Yoon et al., 2014). An improvement of 4.6 and 9.54% was observed during treadmill interval training and resistance exercise, respectively, and this method was found suitable for real-time *f*_R_ monitoring (Yoon et al., 2014). Respiratory wearables based on magnetometers have also shown good agreement, with a bias ± LoA of ~0.2 ± 3 bpm breaths·min^−1^ during moderate walking (McCool et al., 2002). The combination of strain sensors with movement sensors capable of detecting motion artifacts might be an attractive solution for future development of respiratory wearables.

The sound of breathing is used in the clinical field for estimating *f*_R_, but it has received little attention in sport (Peterson et al., 2014). Recording breathing sound during exercise may have some advantages in view of the relatively loud sounds produced, especially during high-intensity. Anecdotally, athletes report monitoring the breathing sounds of their opponents as a gauge of their physical effort during endurance competitions. However, environmental noise can interfere with the quality of the acoustic registration and may explain why little attention has been devoted to breathing sound so far.

It is well-established that ventilation affects the morphology of the ECG signal, and that *f*_R_ can be extracted from the ECG with different techniques (Helfenbein et al., 2014). A few encouraging attempts have also been made to derive *f*_R_ from ECG during cycling exercise (Bailón et al., 2006; Schumann et al., 2016). It is also documented that ventilation affects the PPG signal (Meredith et al., 2012), from which *f*_R_ can be extracted with appropriate computational processing (Charlton et al., 2016). The PPG signal is receiving growing attention in the sports wearable sector because of its simplicity of recording; for instance, it can be obtained from different body sites like the finger, the wrist and the earlobe. Nevertheless, data on the validity of *f*_R_ extracted from the PPG signal during exercise is sparse. In an early attempt made during cycling incremental exercise, motion artifacts prevented a good estimation of *f*_R_ and the error of estimation increased with the increase in exercise intensity (Nakajima et al., 1996). Some of these problems may be overcome with the application of robust filters and appropriate computing techniques (Lee et al., 2011). However, more research is needed to evaluate whether *f*_R_ can be satisfactorily estimated from the ECG or the PPG signal during exercise.

Work on the development of respiratory wearables is likely to increase from a technological point of view (including the computing sector), because a range of sensors and methods can be used to measure *f*_R_. Therefore, we expect growing interest in the development of *f*_R_-based wearables specifically designed for sporting activities, triggered by the understanding of the importance of *f*_R_ for training monitoring. Among the wearables currently available, those measuring chest strain are the most numerous, and their accuracy is generally good. However, the wearability of some of these devices needs to improve before use in monitoring training. Further validation studies are needed to guide sport scientists and practitioners on the choice of the suitable device. Validation studies have generally targeted few exercise modalities (mainly walking and running), and some devices have only been tested during moderate-intensity exercise.

## How should respiratory frequency data be analyzed?

Since we are at an early stage of training monitoring by means of *f*_R_, this section aims to provide some initial guidelines on how to deal with *f*_R_ data. It is important to point out that the variability of *f*_R_ is relatively high if compared to that of other physiological variables like HR (Faude et al., 2017). This is not necessarily a limitation because *f*_R_ is also sensitive to variations in performance induced by a variety of experimental interventions, indicating its relatively high signal-to-noise ratio. However, the variability issue should be considered when analyzing and interpreting *f*_R_ data. A breath-by-breath *f*_R_ dataset should be filtered for errant breaths (i.e., values resulting after coughs, sighs, swallows, etc.), as commonly performed for gas exchange analysis (Lamarra et al., 1987). Subsequently, data can be interpolated to 1-s intervals and bin averaged according to experimental or practical needs. Due to the inherent variability of *f*_R_, the maximal value of *f*_R_ (*f*_Rmax_) should not be taken from breath-by-breath values but from an average of no <10 s. For the same reason, average values should be displayed real time during training activities rather than breath-by-breath values.

**Figure 2:**
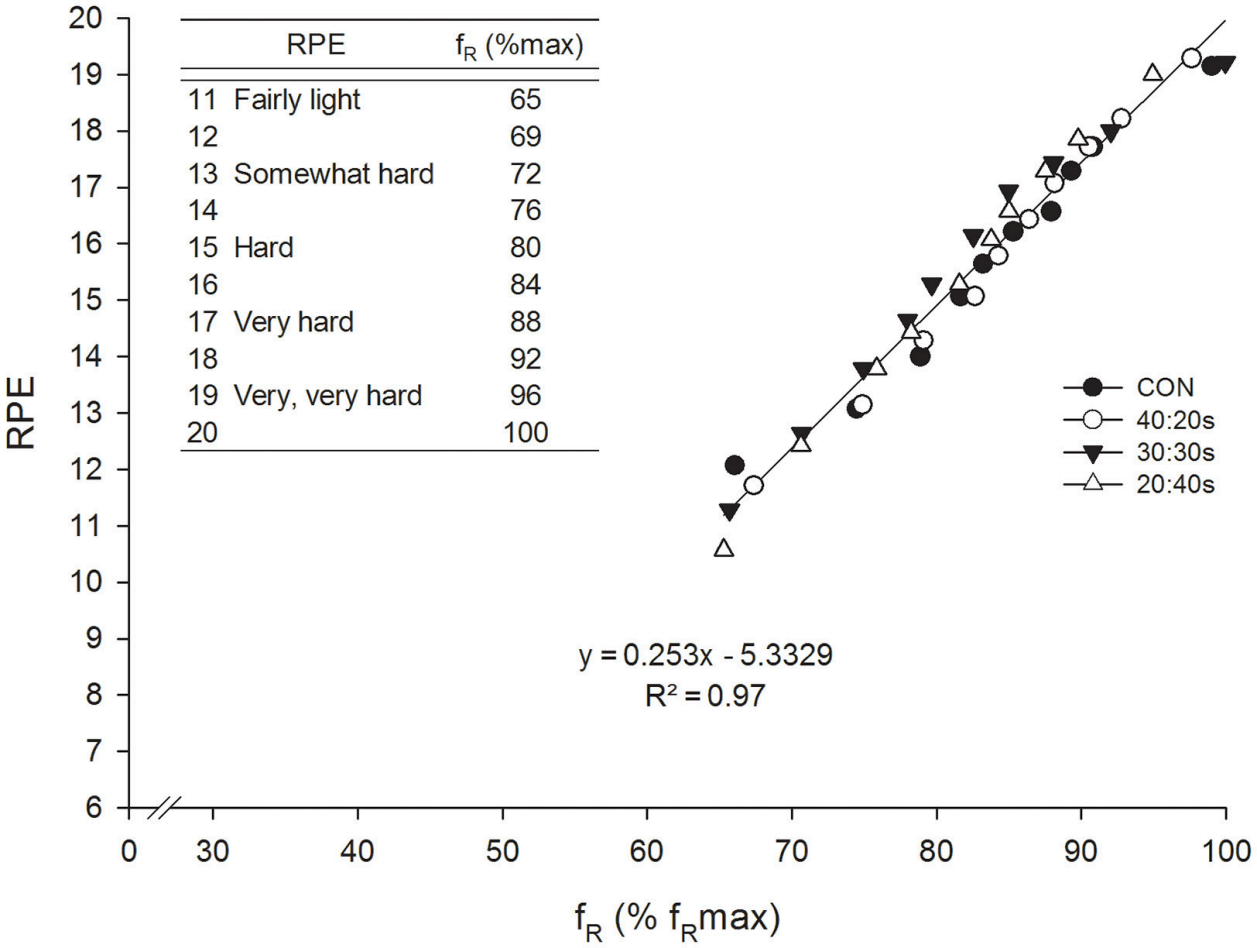
Correlation between RPE and *f*_R_ normalized to *f*_Rmax_ during a continuous (CON) and three different HIIT trials (40:20 s, 40 s work 20 s rest; 30:30 s, 30 s work 30 s rest; 20:40 s, 20 s work 40 s rest) matched for effort and exercise duration (30 min). The linear regression results from pooling together data from the four trials. The regression equation of the correlation obtained was used to associate *f*_R_ normalized to *f*_Rmax_ with the 6–20 RPE scale (upper left corner of the chart). This was done in order to favor the interpretation of *f*_R_-values obtained during exercise. Reproduced from Nicolò et al. (2014a).

The *f*_Rmax_ reached during maximal effort exercise is similar across different exercise paradigms and durations (Kift and Williams, 2007; Nicolò et al., 2014a,b, 2016a, 2017b), with few extreme exceptions (Nicolò et al., 2015). Therefore, different maximal exercise protocols appear to be suitable for measuring *f*_Rmax_. It is convenient to normalize *f*_R_ to *f*_Rmax_ to develop prescription and monitoring strategies that can be generalized, since there is relatively high variability in *f*_Rmax_ across different individuals, and the factors determining this variability are not well-understood. The first attempt to interpret *f*_R_ data normalized to *f*_Rmax_ was made by Nicolò et al. (2014a). They found a strong correlation between *f*_R_ and RPE with similar values across a continuous and three different HIIT trials matched for effort and exercise duration. Therefore, values from the four trials were considered together, and the regression equation of the correlation obtained was used to associate *f*_R_ normalized to *f*_Rmax_ with the well-known 6–20 RPE scale (Figure 2). For instance, a value of 80% *f*_Rmax_ approximately corresponded to an effort perceived as hard, and a value of 88% *f*_Rmax_ to an effort perceived as very hard, with clear implications for training prescription and monitoring. Indeed, *f*_R_ is an objective variable that can be measured continuously during exercise, while RPE is a subjective variable which can only be collected at discrete points in time. This approach could be improved further by normalizing *f*_R_ to the range of possible *f*_R_-values available (from *f*_R_ measured at rest to *f*_Rmax_), in a similar manner to the formula used to obtain the HR reserve (Karvonen and Vuorimaa, 1988). This normalization procedure could be used to provide objective real-time feedback on physical effort, with values conveniently ranging from 0 to 100. A real-time feedback could also allow athletes to voluntary alter their breathing pattern as allegedly advised by some coaches, although the potential benefit of this practice is uncertain.

Different approaches may be used to synthesize *f*_R_ data from one or more training sessions. Unlike for HR, average *f*_R_ is similar across maximal-effort training sessions differing in the HIIT format of exercise or duration (Nicolò et al., 2014a, 2016a, 2017b). Therefore, average *f*_R_ may provide a simple preliminary description of the overall physical effort of a training session. However, more comprehensive analyses are required to fully examine the potential of *f*_R_ data. Two promising analyses conceived to analyze large datasets are the training distribution and the training concentration profiles described by Passfield and Hopker (2017). The training distribution profile shows the total session time spent above the reference *f*_R_-value (which can be interpreted as the reference level of effort), which assumes every possible value (Figure 1D). The training concentration profile is a concentration curve (i.e., the derivative of the distribution curve), which shows the cumulative time spent training at each *f*_R_-value (effort level) (Figure 1E). *f*_R_ distribution and *f*_R_ concentration profiles would therefore provide a breakthrough in understanding training effort, which is currently summarized by a single session value of RPE.

## Conclusion

In this perspective article, we aimed to present scientific evidence indicating the importance of monitoring *f*_R_ during training, and to propose possible methodologies and wearable sensors currently available to measure *f*_R_ in the field. We also provided indications on how to analyze and interpret *f*_R_ data. This is expected to benefit athlete training monitoring and the advancement of applied research in this area of sports science, and to stimulate the development and use of respiratory wearables specifically designed for sporting activities. That of *f*_R_ represents a good example of how wearable sensor development should follow athlete's needs and be informed by scientific findings.

## Author contributions

All authors (AN, CM, and LP) contributed to the conception and design of the work, drafted the work or revised it critically for important intellectual content and approved the final version of the manuscript. All authors (AN, CM, and LP) agree to be accountable for all aspects of the work in ensuring that questions related to the accuracy or integrity of any part of the work are appropriately investigated and resolved.

### Conflict of interest statement

The authors declare that the research was conducted in the absence of any commercial or financial relationships that could be construed as a potential conflict of interest.
